# Acute myocardial infarction in a 41-year-old woman due to elevated factor VIII: a case report

**DOI:** 10.11604/pamj.2021.38.207.28011

**Published:** 2021-02-24

**Authors:** Falmata Laouan Brem, Hammam Rasras, Noha El Ouafi, Zakaria Bazid

**Affiliations:** 1 Department of Cardiology, Mohammed VI University Hospital, Mohammed First University, Oujda, Morocco,; 2 Mohammed VI University Hospital, Epidemiological Laboratory of Clinical Research and Public Health, Oujda, Morocco

**Keywords:** Acute myocardial infarction, coagulation factor VIII, thrombophilia, coronary artery thrombosis, case report

## Abstract

Myocardial infarction is a life-threatening emergency with a high mortality rate. A high plasma level of factor VIII is an established risk for both arterial and venous thrombotic events. In this mini-review, we report the case of a 41-year-old woman without cardiovascular risk factors or a previous history of thrombotic events, admitted for ST-elevation myocardial infarction, in whom coronary angiography showed a thrombotic occlusion in the left anterior descending artery. The patient underwent primary percutaneous coronary intervention (PCI), with GPIIB-IIIA antagonist, then, a pre-dilation with a semi-compliant balloon-catheter, followed by implantation of 2 stents. The etiological assessment revealed a high level of coagulation factor VIII (FVIII). She underwent anticoagulation therapy (with acenocoumarol) with well-controlled international normalised ratio (INR).

## Introduction

Thrombotic disorders, especially in developing countries, are well-known causes of morbidity and mortality [1]. Procoagulant factor VIII (FVIII) acts as a cofactor for factor IX and yields an important role in the coagulation cascade [2]. A high plasma level of factor VIII is an established risk for both arterial and venous thrombotic events [3-5]. However, the prevalence of elevated FVIII in arterial thrombosis still undetermined. Treatment includes that of myocardial infarction by revascularization of the infarcted territory and that of the cause (hypercoagulability state) with a therapeutic dose of anticoagulation therapy which has shown its effectiveness [6].

## Patient and observation

**Figure 1:**
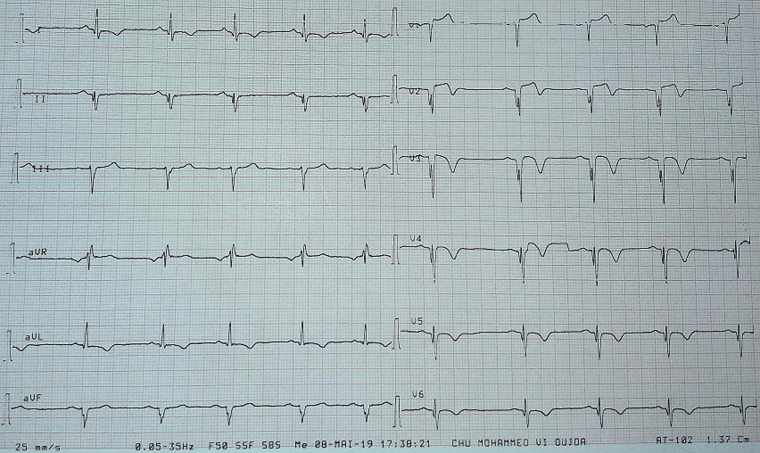
electrocardiogram (EKG)

**Figure 2:**
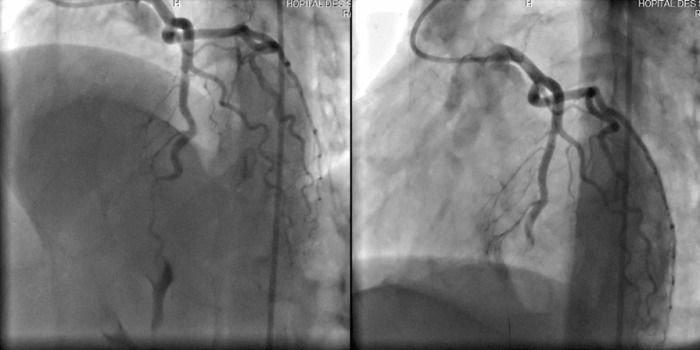
coronary angiography, showing a thrombus in the middle segment of the left anterior descending artery

**Figure 3:**
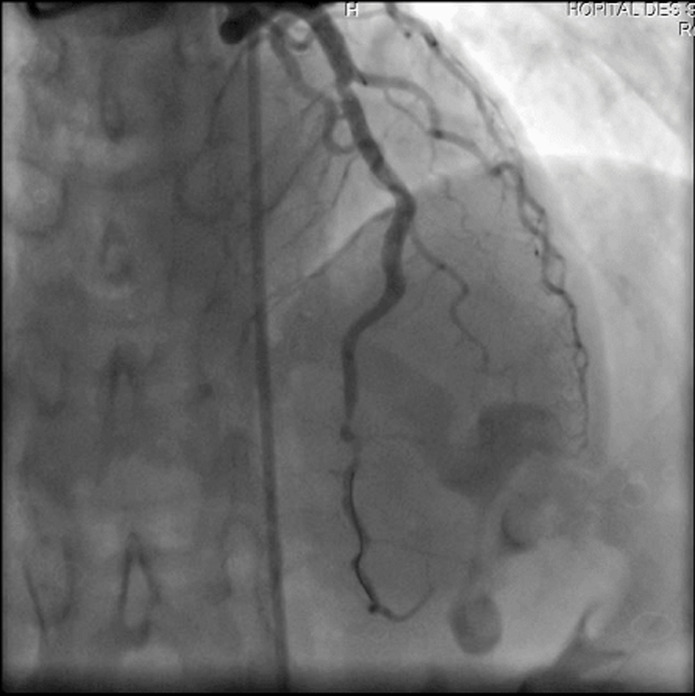
coronary angiography showing percutaneous coronary intervention (PCI) of the left anterior descending artery with implantation of 2 stents

A 41-year-old woman with no history of thrombotic events and without cardiovascular risk factors was admitted for an anterior ST-elevation myocardial infarction (STEMI) (Figure 1). The physical examination was without abnormalities. The patient received pharmacological measures (acetylsalicylic acid - ASA 300mg, clopidogrel 600mg and low-molecular-weight heparins (LMWHs)). Transthoracic echocardiography (TTE) showed hypokinesia of the anteroseptal wall and the apex with a good systolic function of the left ventricle (LV) left ventricular ejection fraction (LVEF) at 51%. Coronary angiography showed a thrombotic occlusion in the left anterior descending artery (Figure 2). The patient underwent primary percutaneous coronary intervention (PCI), with GPIIB-IIIA antagonist, then a pre-dilation with a semi-compliant balloon-catheter, followed by implantation of 2 stents (Figure 3). The etiological assessment, including a lipid profile, glycated hemoglobin, thyroid function test, homocysteine, fibrinogen, protein S, protein C, antithrombin III, antiphospholipid antibodies showed a normal result. Factor V levels were slightly elevated (128% (normal range: 70% - 120%)), with high levels of F VIII controlled two times (333% (normal range: 70% - 120%)). She underwent anticoagulation therapy (with acenocoumarol) with well-controlled INR.

## Discussion

The Leiden thrombophilia study was the first study to report elevated FVIII as a behind-agent of venous thromboembolism [7]. Then, it was broadly reported in the literature. However, as an etiology of arterial thrombosis, it was little-reported. The first presentation of this rare association was in 1962, which was between high levels of FVIII and coronary artery disease [8]. The implication of FVIII in the pathogenesis of arterial thrombosis still unclear despite important progress [7]. Some authors suggested the implication of genetic in the pathogenesis of arterial thrombosis of factor VIII [9].

The PLAT study (Progretto Lombardo Atero-Trombosi) reported a high level of FVIII as an independent predictor in vascular diseases [10], in myocardial infarction, stroke and peripheral ischemia. In addition, there have been a few reports of acute myocardial infarction that occurred during the infusion of recombinant factor VIII in patients with hemophilia [11-13]. The diagnostic approach of arterial thrombosis is less clear than venous thrombosis. The incidence of acute myocardial infarction reported in patients under 45 years is 5-10% and many of them have none of the common cardiovascular or thrombotic risk factors [14]. Therefore, hypercoagulability state testing appears to be more important in these patients. Furthermore, in these cases, testing of the patient´s relatives is mightily advised [9].

The therapeutic approach focuses on two parties. The first is emergency case management, which includes early revascularization to escape the dread of life-threatening ischemia complications associated with a high mortality rate. Then comes a major discussion about anticoagulation against the hypercoagulability state. As part of the secondary prevention of the development of the thrombi formation, anticoagulants (warfarin and other vitamin K antagonists) showed their effectiveness in patients with myocardial infarction and other arterial thromboses such as atrial fibrillation and peripheral arterial disease [6] and here, we suggested that it may be valuable to screen for these thrombotic disorders to reduce recurrence [15] and severe complications such as catastrophic thrombotic syndrome (thrombotic storm) [16].

## Conclusion

Previous data, primarily mainly epidemiological, suggest that a high plasma level of factor VIII is an independent risk factor for venous and arterial thrombosis. However, further studies are needed to assess the involvement of an elevated factor VIII in the coronary thrombus formation leading to acute myocardial infarction.
